# Spatially resolved mapping of phase transitions in liquid-crystalline materials by X-ray birefringence imaging†
†Electronic supplementary information (ESI) available. See DOI: 10.1039/c8sc05285a


**DOI:** 10.1039/c8sc05285a

**Published:** 2019-01-02

**Authors:** Yating Zhou, Rhian Patterson, Benjamin A. Palmer, Gregory R. Edwards-Gau, Benson M. Kariuki, N. S. Saleesh Kumar, Duncan W. Bruce, Igor P. Dolbnya, Stephen P. Collins, Andrew Malandain, Kenneth D. M. Harris

**Affiliations:** a School of Chemistry , Cardiff University , Park Place , Cardiff CF10 3AT , Wales , UK . Email: HarrisKDM@cardiff.ac.uk; b Diamond Light Source , Harwell Science and Innovation Campus , Didcot , Oxfordshire OX11 0DE , England , UK; c Department of Structural Biology , Weizmann Institute of Science , Rehovot , 760001 , Israel; d Department of Chemistry , University of York , Heslington , York YO10 5DD , England , UK

## Abstract

The X-ray Birefringence Imaging (XBI) technique, first reported in 2014, is a sensitive method for spatially resolved mapping of the local orientational properties of anisotropic materials. We report the first application of the XBI technique to characterize molecular orientational ordering in a liquid crystalline material, demonstrating significant potential for exploiting XBI measurements to advance structural understanding of liquid crystal phases.

## Abstract 2

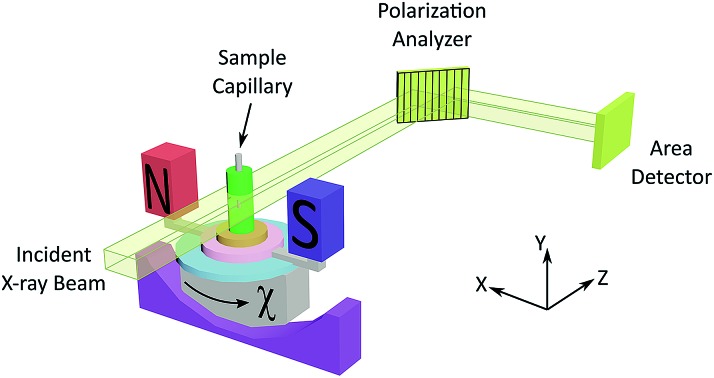
X-ray Birefringence Imaging is shown to be a sensitive method for spatially resolved mapping of molecular orientations in liquid-crystalline materials.

## 

In many scientific disciplines, the phenomenon of optical birefringence^1^ is used as a basis for studying structural anisotropy of materials through the use of the polarizing optical microscope,^2^,^3^ including the widespread application of this technique in the characterization of liquid-crystalline phases.^4^,^5^ While optical birefringence is widely exploited in this way, the opportunity to study birefringence of anisotropic materials using linearly polarized X-rays^6^–^14^ has remained remarkably neglected. Indeed, the first definitive demonstration of X-ray birefringence was reported only recently^10^ for a model material that was shown to exhibit essentially ideal birefringence behaviour at X-ray energies near the Br K-edge. While optical birefringence depends on the overall symmetry properties of a material, X-ray birefringence (when studied using an X-ray energy corresponding to an absorption edge of an element in the material) is sensitive to the *local* orientational properties of individual molecules and/or bonds.

The phenomenon of X-ray birefringence is closely related to the phenomenon of X-ray dichroism,^15^–^19^ both of which concern the interaction of linearly polarized X-rays with anisotropic materials. Specifically, these phenomena relate to the way in which X-ray absorption (in the case of dichroism) and the real part of the complex refractive index (in the case of birefringence) depend on the orientation of the material relative to the direction of linear polarization of the incident X-ray beam. Although X-ray dichroism and X-ray birefringence give rise to different effects on the propagation of linearly polarized radiation through a material, they are related by a Kramers–Kronig transform^20^ and the two phenomena depend on the same structural and symmetry properties of the material.

The ability of X-ray birefringence to yield insights into molecular orientational properties was first exploited^10^ for accurate determination of bond orientations and for establishing *changes* in molecular orientational distributions associated with order–disorder phase transitions in solids.^11^ However, these early X-ray birefringence studies used a narrowly focused incident X-ray beam and did not provide spatially resolved mapping of X-ray birefringence across the material. Subsequently, a new technique called X-ray Birefringence Imaging (XBI) was developed^21^ to allow X-ray birefringence data to be measured in a spatially resolved manner. In many respects, the XBI technique represents the X-ray analogue of the polarizing optical microscope, and it has been shown to be a sensitive technique for imaging the local orientational properties of anisotropic materials, allowing orientationally distinct domain structures to be identified and yielding information on the size, spatial distribution, temperature dependence and orientational relationships of such domains.

The experimental set-up for XBI (see Fig. 1 and Experimental) uses a large (*e.g.* 4 mm × 4 mm) unfocused incident linearly polarized X-ray beam, with energy tuned to an absorption edge of an element in the material. The X-rays transmitted through the sample are interrogated using a diffraction-based polarization analyzer (set at a Bragg angle as close as possible to 2*θ* = 90°), and the resultant X-ray intensity is recorded in a spatially resolved manner using an area detector. The XBI technique was first demonstrated^21^ using incident polarized X-rays at the Br K-edge for a single crystal of the 1-bromoadamantane/thiourea inclusion compound^18^ in which the C–Br bonds of all 1-bromoadamantane guest molecules are aligned parallel to each other within the one-dimensional tunnels of the thiourea host structure. The results demonstrated uniaxial behaviour, and confirmed that the *X-ray optic axis* corresponds to the C–Br bond orientation.^22^ XBI studies of an order–disorder phase transition in a single crystal of the bromocyclohexane/thiourea inclusion compound^21^ revealed the existence of orientationally distinct domains in the low-temperature phase, yielding new insights into the domain sizes and the orientational relationship between domains. And further XBI studies^23^ demonstrated that, for materials undergoing anisotropic molecular dynamics, the effective X-ray optic axis is the *time-averaged* resultant of the orientational distribution of the C–Br bonds.

**Fig. 1:**
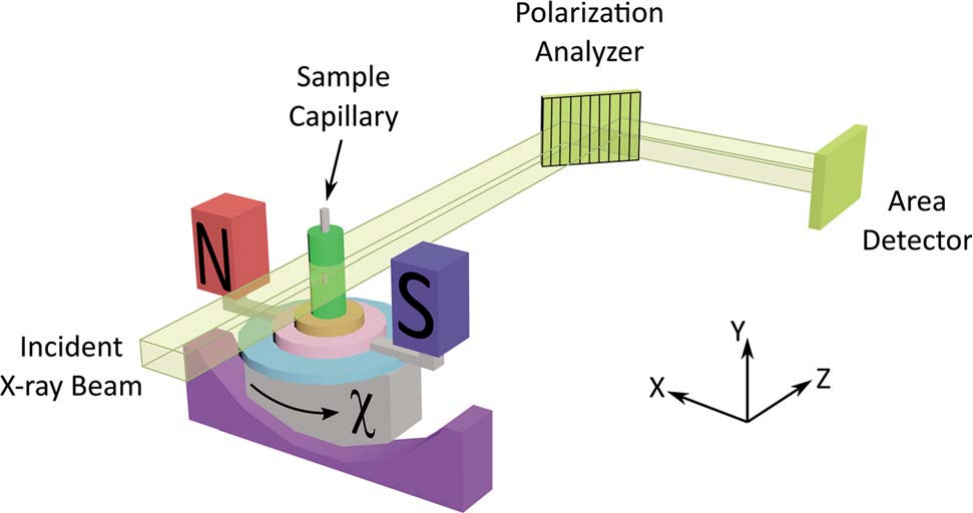
Experimental set-up for XBI studies of liquid crystal samples oriented in an applied magnetic field. The incident X-ray beam propagates along the *z*-axis and is polarized almost entirely linearly along the *x*-axis. The angle between the axis of the magnetic field and the direction of linear polarization of the incident X-ray beam (horizontal) is denoted *χ* (corresponding to rotation about the *z*-axis).

As X-ray birefringence is sensitive to *local* molecular orientational properties, there is no requirement for the sample to be crystalline, and the XBI technique may be applied to probe the distribution of molecular orientations in any anisotropic material. In this paper, we report the first application of XBI to study molecular orientational ordering in liquid-crystalline phases, using an experimental assembly designed specifically for the measurement of XBI data for liquid crystals aligned in an applied magnetic field. In this set-up (Fig. 1 and Experimental), the sample cell is mounted on the goniometer of the synchrotron beamline, allowing the orientation of the sample to be changed relative to the direction of polarization (horizontal) of the linearly polarized incident X-ray beam. The sample cell includes a strong magnetic field (Sm–Co magnet; field strength *ca.* 1.0 T) to align the liquid crystal phases and a variable temperature capability, controlled by passing an electric current through the graphite outer sample holder (Fig. 2), to which a thermocouple is attached for temperature measurement. In this set-up, the sample orientation is specified by the angle *χ* (see Fig. 1 and 2), which defines the angle between the applied magnetic field direction (the expected axis of molecular alignment in the liquid crystal phases) and the orientation of the linearly polarized incident X-ray beam (horizontal). In the set-up used in the present work, the value of *χ* may be varied in the range *χ* = 45° to *χ* = –45°.

**Fig. 2:**
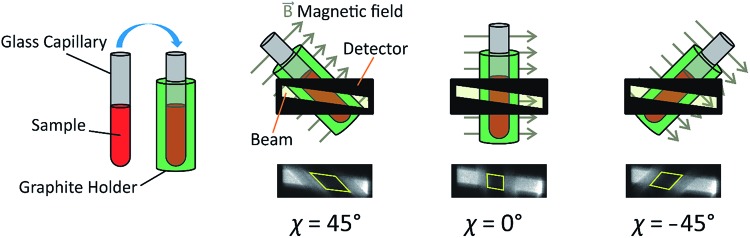
Schematic of the sample assembly in XBI studies of liquid crystal samples. The sample is placed inside a glass capillary, which is inserted inside an outer sample holder made from graphite. The magnetic field is perpendicular to the long axis of the capillary and perpendicular to the direction of propagation of the incident X-ray beam. The angle between the axis of the magnetic field and the direction of linear polarization of the incident X-ray beam (horizontal) is denoted *χ*. The region of each XBI image corresponding to X-rays transmitted through the sample is defined by the yellow parallelogram (in the images shown, the sample is an isotropic liquid phase).

The material selected for study was 4′-octyloxy-[1,1′-biphenyl]-4-yl-4-bromobenzoate (Scheme 1).^24^ As XBI data recorded at the Br K-edge are sensitive to the orientational distribution of the C–Br bonds, the presence of the terminal C–Br bond in this compound provides a basis for establishing the distribution of molecular orientations in the liquid crystal phases from analysis of the XBI data. This compound, which melts on heating at 151 °C, is reported^24^ to have the following phase sequence on cooling:Iso · 216 °C · N · 215 °C · SmA · 154 °C · SmBwhere we use the common abbreviations: Iso, isotropic liquid; N, nematic; SmA, smectic A; SmB, smectic B. The presence of the rather transient nematic phase offers the possibility for alignment by an applied magnetic field on cooling and it would reasonably be expected that the terminal C–Br bond would be coincident with, or at least extremely close to, the director (**n**). Because the experimental set-up (Fig. 1) allows the sample orientation to be varied with respect to the incident beam polarization, then the magnetic alignment promises to allow good-quality orientational information to be extracted from the XBI data.

**Scheme 1:**
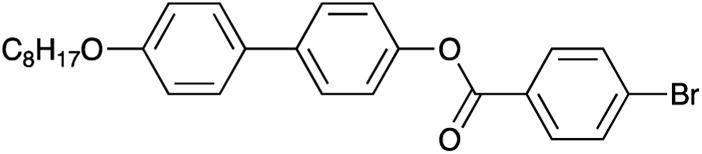


Differential scanning calorimetry (DSC) carried out in the present work (Fig. 3) gives phase transition temperatures (Iso · 216 °C · N · 214 °C · SmA · 153 °C · SmB) in close agreement with those (see above) reported by Takeda *et al.*,^24^ which were derived by optical microscopy. The observed temperature of the transition from the smectic B phase to the crystalline phase depends on the experimental conditions as a result of supercooling.

**Fig. 3:**
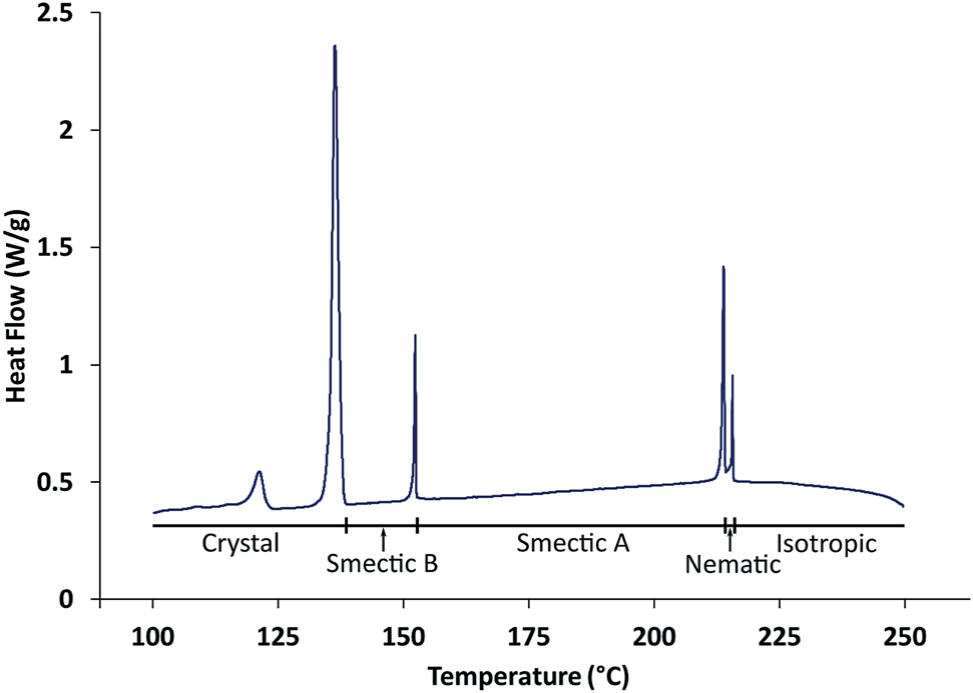
DSC data recorded on cooling from the isotropic liquid phase at 1 °C min^–1^. The transition at 123 °C is a solid–solid phase transition within the crystalline phase.

Fig. 4 shows selected images from the XBI data recorded at 220 °C (isotropic liquid; Fig. 4a), 214 °C (nematic phase and some isotropic liquid as a consequence of a small temperature gradient; Fig. 4b) and 184 °C (smectic A phase; Fig. 4c) as a function of the orientation of the sample relative to the incident X-ray beam, which was linearly polarized in the horizontal direction and tuned to the Br K-edge (a more comprehensive set of XBI images from this experiment is shown in Fig. S1 in ESI†). Each XBI image shown in Fig. 4 represents a spatially resolved map of transmitted X-ray intensity for a specific orientation of the sample specified by angle *χ* (defined in Fig. 1 and 2). The magnetic field was kept in the plane (*xy*-plane) perpendicular to the propagation direction (*z*-axis) of the incident X-ray beam. The angle *χ* denotes rotation of the magnetic field around the *z*-axis, and thus specifies the direction of molecular alignment of the liquid crystal phases relative to the direction of linear polarization of the incident X-ray beam. For *χ* = 0°, the magnetic field is horizontal (parallel to the *x*-axis).

**Fig. 4:**
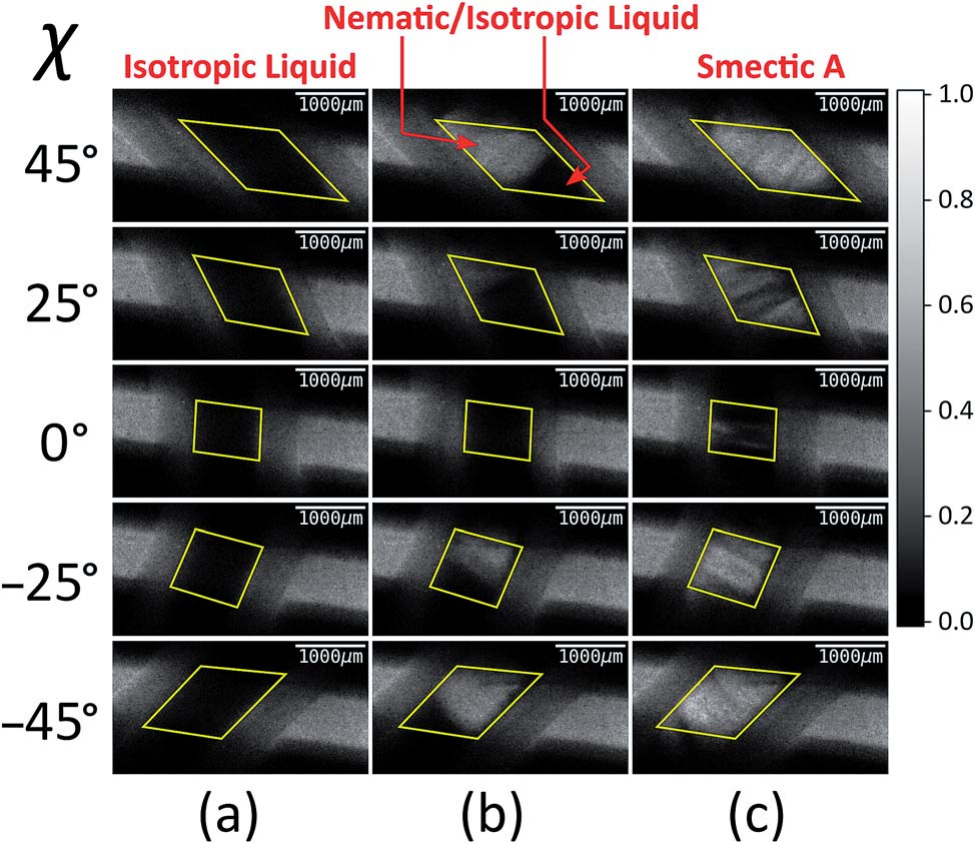
Selected XBI images recorded as a function of the orientation of the applied magnetic field (defined by angle *χ*) at the following temperatures: (a) 220 °C (isotropic liquid), (b) 214 °C (both nematic and isotropic liquid phases are present on account of a temperature gradient), and (c) 184 °C (smectic A phase). The scale of normalized X-ray intensity is shown on the right-hand side. The region of each XBI image representing the sample is defined by the yellow parallelogram.

For the isotropic liquid phase, the XBI images (Fig. 4a) are uniformly dark for all sample orientations, with no variation in X-ray intensity as a function of sample orientation (Fig. 5) and hence zero birefringence. These observations are fully consistent with an isotropic distribution of C–Br bond orientations in this phase.

**Fig. 5:**
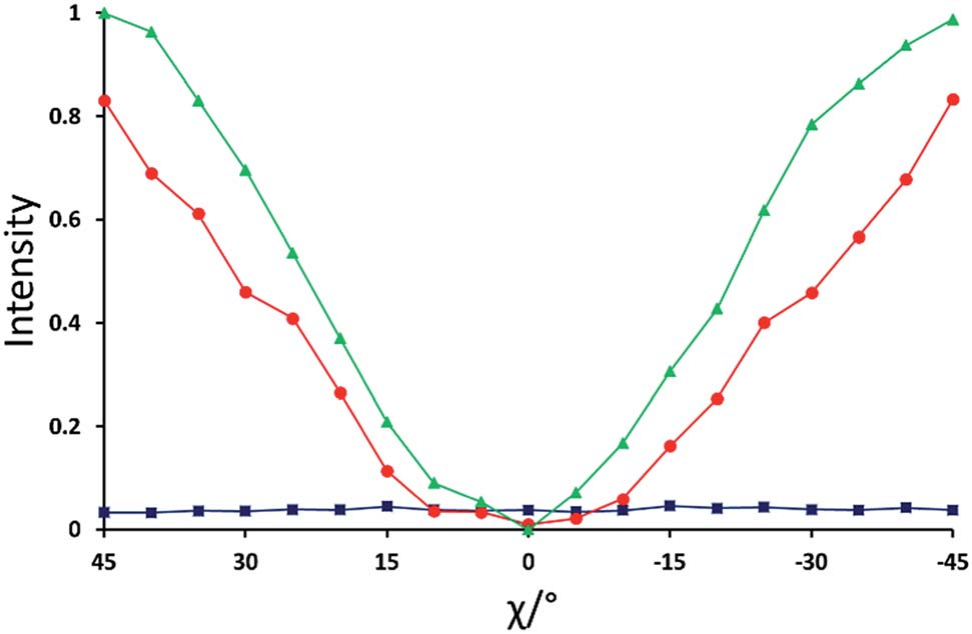
Normalized X-ray intensity as a function of *χ* for the XBI data recorded at: 220 °C (blue; isotropic liquid); 214 °C (red; nematic phase), and 184 °C (green; smectic A phase). Selected XBI images from the same experiment are shown in Fig. 4, and a more complete set of images are shown in Fig. S1.† X-ray intensity was measured as the average intensity per pixel across a selected area of the sample region in the XBI image. At 214 °C, the sample comprises a region of nematic phase and a region of isotropic liquid (see Fig. 4b), and the intensity was measured within the region of the image known to represent the nematic phase. Measured intensities *I*_meas_ were normalized to give a value in the range 0 ≤ *I*^*N*^ ≤ 1, with *I*^*N*^ = (*I*_meas_ – *I*_min_)/(*I*_max_ – *I*_min_). Here, *I*_max_ and *I*_min_ are the highest and lowest measured intensities in the entire set of data (*i.e.* for all XBI images recorded at the three temperatures shown).

Starting from the isotropic liquid, the sample was oriented at *χ* = 45° and cooled in small increments in the temperature region near the phase transition to the nematic phase, until the first change in X-ray intensity was observed in the XBI data.^25^ At 214 °C, the XBI image recorded at *χ* = 45° (top image in Fig. 4b) clearly contains both a bright region (upper left) and a dark region (bottom right), representing the first temperature during cooling at which there was evidence of the orientationally ordered nematic phase. From the changes in the XBI data as a function of *χ* (Fig. 4b), the region identified as the nematic phase clearly exhibits significant birefringence. In contrast, the other region of the sample remains dark in the XBI images at all values of *χ* and is assigned as the isotropic liquid. The presence of both nematic and isotropic liquid phases is a consequence of a temperature gradient within the sample holder.^26^

For the nematic phase, the variation in X-ray intensity measured from the XBI images as a function of *χ* is shown in Fig. 5, demonstrating (at least within experimental errors) the type of sinusoidal behaviour^27^ expected for a uniaxial system in which the optic axis is parallel to the magnetic field direction (*i.e.* with intensity minimum at *χ* = 0° and intensity maxima at *χ* = 45° and *χ* = –45°). As the effective optic axis for XBI studies at the Br K-edge is dictated by the resultant direction of alignment of the C–Br bonds, the X-ray birefringence behaviour for the nematic phase is clearly interpreted in terms of a high degree of orientational ordering of the molecules, with a resultant C–Br bond orientation effectively parallel to the magnetic field.

The XBI behaviour for the smectic A phase (184 °C; Fig. 4c) is very similar to the nematic phase, exhibiting significant variation in X-ray intensity as a function of *χ* with minimum brightness at *χ* = 0° and maximum brightness at *χ* = 45° and *χ* = –45°. As shown in Fig. 5, the X-ray intensity again exhibits a sinusoidal variation as a function of *χ*. Significantly, the maximum intensity is higher than the maximum intensity for the nematic phase, indicating that, as expected for a more ordered phase with partial translational ordering, the smectic A phase has a higher degree of orientational ordering of the C–Br bonds (*i.e.* a narrower orientational distribution of the C–Br bonds) in the direction of the applied magnetic field.

In this experiment, XBI data were also recorded at selected lower temperatures, but did not include a measurement that could be unambiguously identified as the smectic B phase.^28^ Instead, reliable intensity information for the smectic B phase has been obtained from XBI images recorded in experiments with fixed sample orientation discussed below. XBI images recorded at temperatures that are clearly within the crystalline phase (Fig. S2 in ESI†) indicate that the sample exists in multi-crystal domains. The X-ray intensity for the crystalline phase is significantly lower than that for the smectic A phase, which may be a consequence of the X-ray beam passing through several domains with different orientations on its path through the polycrystalline sample and/or may reflect a lower resultant degree of alignment of the C–Br bonds in this phase (for example, if there are two or more distinct orientations of the C–Br bonds in the unit cell; we note, however, that the crystal structure of this material has not been reported).

The type of experiment described above, in which XBI data were measured by changing the sample orientation by *χ*-scans at selected fixed temperatures was found to be problematic in the case of liquid-crystalline phases, as it was often observed in such experiments that the domain structure could occasionally change suddenly and unpredictably on changing the value of *χ*, reflecting the fluid nature of these phases under gravity. Under such circumstances, it is difficult to extract reliable information on the characteristic differences in X-ray intensity between the different liquid crystal phases. Instead, we have found that a much more reliable protocol for exploring the changes in the degree of ordering in the liquid crystal phases as a function of temperature is to record the XBI images with the orientation of the applied magnetic field *fixed* at *χ* = 45° while scanning through the temperature range of interest. The results from an experiment of this type are shown in Fig. 6, in which the XBI images were recorded on decreasing the temperature from 218 °C (*i.e.* starting in the isotropic liquid phase) to 108 °C at a rate of 1 °C min^–1^, with the XBI images recorded continuously during the cooling process (time per image, 5 s). At the highest temperature, which is above the clearing temperature, the intensity is very low as a result of the isotropic orientational molecular distribution in the isotropic liquid phase. On decreasing the temperature, the intensity increases substantially between 216 °C and 205 °C, representing the transition from the isotropic liquid into orientationally ordered phases (from Iso → N → SmA). The upper part of Fig. 6 shows the evolution of the X-ray intensity measured from the XBI images as a function of temperature. In the range between 216 °C and 205 °C, the data show a ‘first-order’ change in intensity at the clearing point as the nematic phase forms, after which there is a small inflection over the approximate temperature range 215 °C to 211 °C (corresponding to the intensity range from *ca.* 0.2 to 0.4). The fact that the sharp rise in intensity between 216 °C and 205 °C covers a significantly wider temperature range than the Iso → N → SmA events observed by DSC and optical microscopy may reflect a combination of the temperature gradient across the sample plus the kinetics of alignment in the presence of the magnetic field. On decreasing the temperature within the SmA phase, the intensity shows a gradual increase until a visible transition into the SmB phase is observed from a further sharp (although relatively small) increase in intensity, followed by a significant decrease in intensity upon crystallization.

**Fig. 6:**
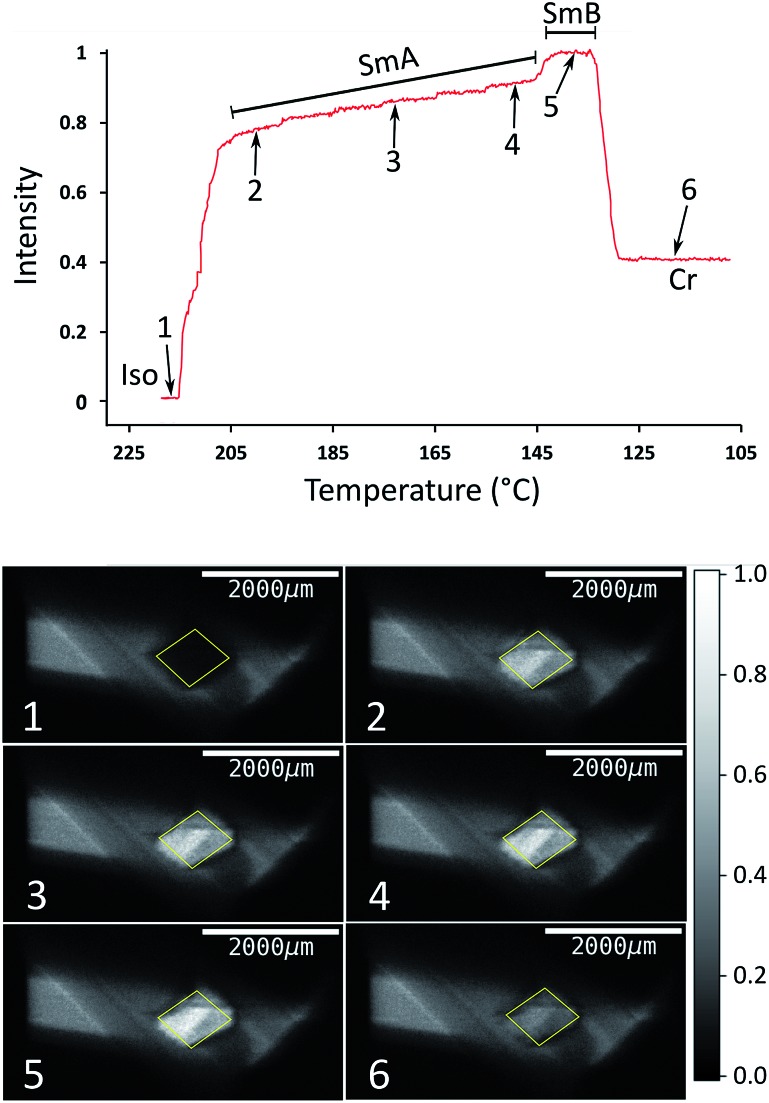
Top: Normalized X-ray intensity in XBI images recorded on decreasing temperature from 218 °C to 108 °C at 1 °C min^–1^, with the orientation of the applied magnetic field fixed at *χ* = 45°. The phase transitions are associated with abrupt changes in intensity, as evident from comparison to the DSC data (Fig. 3); the region corresponding to the Iso → N → SmA transition sequence is discussed in the text. Bottom: XBI images recorded at different stages of the cooling process (the specific temperature and measured intensity for each XBI image, numbered from 1 to 6, is identified from the plot at the top). The region of each XBI image representing the sample is defined by the yellow parallelogram.

The order in liquid crystal phases can be quantified by a series of order parameters, of which the most commonly used is the orientational order parameter, *S* = = 〈½(3 cos½(3 cos^2^ *θ* – 1) − 1)〉, where , where *θ* is the angle between the director and the individual long molecular axes. The value of *S* is often determined by measurement of the optical birefringence (*S* is proportional to the birefringence). As an example, Fig. S3† shows the evolution of the refractive index in a material that has the same phase transition sequence as the compound under study here, albeit with different phase ranges.^29^ The behaviour in Fig. S3† reveals a steep initial increase in birefringence (and hence in the order parameter) that flattens as the sample is cooled away from the clearing point, which is qualitatively the same as the evolution of X-ray intensity with decreasing temperature in Fig. 6. Clearly, the X-ray intensity in XBI data is related to the order parameter *S*, and while our interpretations of the changes in X-ray intensity as a function of temperature have invoked this relation at a qualitative level, our future research will include the derivation of a more quantitative framework for determining values of order parameters from XBI data.

The results reported in this paper represent the first experiments to study the orientational ordering of liquid crystal phases using the XBI technique, and demonstrate clearly the capability of the method to identify the direction of preferential molecular alignment in these phases and to establish, at least qualitatively, the relative degree of molecular orientational ordering in the different liquid crystal phases observed. The type of experiment reported in Fig. 6, involving the measurement of XBI images as a detailed function of temperature for a fixed sample orientation, provides a clear indication of the occurrence of phase transitions between the liquid crystal phases as well as indicating the changes in the degree of orientational ordering associated with these phase transitions, and we anticipate that this type of temperature-resolved XBI measurement will prove to be particularly useful in the liquid crystals field. Our future research will extend these initial studies to encompass a significantly wider range of types of liquid-crystalline material, including those containing metal atoms that may be utilized as the X-ray absorbing element in the XBI measurements. Our plans to further develop the sample cell for carrying out XBI measurements on liquid crystal phases (particularly focusing on improving the accuracy of temperature control and reduction of temperature gradients) are likely to allow structural information of a more quantitative nature to be derived from our future studies.

## Experimental

Synthesis of 4′-octyloxy-[1,1′-biphenyl]-4-yl-4-bromobenzoate (Scheme 1) was carried out using the procedure reported previously.^24^ Phase transitions were checked by optical microscopy (Olympus BX50 polarizing microscope equipped with a Linkam LTS350 heating stage, Linkam LNP2 cooling pump and Linkam TMS92 controller) and by DSC (TA Instruments Q100 differential scanning calorimeter). For the DSC measurements, the powder sample was loaded into a hermetically sealed aluminium pan and heated to 250 °C to produce the liquid phase; DSC data were then recorded on cooling to 100 °C at 1 °C min^–1^ or 10 °C min^–1^.

All XBI experiments were carried out on beamline B16 at Diamond Light Source with the sample mounted on a five-circle, vertical-scattering, Huber Eulerian diffractometer.^30^ The incident X-ray energy was 13.490 keV, corresponding to the Br K-edge. In the experimental set-up for XBI (Fig. 1), a wide unfocused linearly-polarized incident X-ray beam is incident upon the sample; the X-rays transmitted through the sample undergo Bragg diffraction at the polarization analyzer, and the intensity of the X-ray beam diffracted from the polarization analyzer is recorded in a spatially resolved manner using an area detector. In this work, the beam dimensions were defined by slits of 4 mm (vertical) and 4 mm (horizontal), and the polarization analyzer was a Si(555) crystal. For the *χ*-scans at fixed temperature (Fig. 4, S1 and S2†), the area detector was an SCMOS X-ray camera from Photonic Science Ltd (pixel size, 3.25 μm; image dimensions, 1920 × 1080 pixels) and the time to record each XBI image was 2 s. For the XBI images recorded as a function of temperature at fixed sample orientation (Fig. 6), the area detector was a 12-bit CCD miniFDS camera from Photonic Science Ltd (pixel size, 6.5 μm; image dimensions, 1392 × 1040 pixels) and the time to record each XBI image was 5 s. For the experimental set-up used in this work, the spatial resolution of the XBI images was *ca.* 10 μm. The spatial resolution of the XBI images in the vertical direction (*ca.* 13 μm) is limited by the resolution of the CCD-based detector. The spatial resolution in the horizontal direction is limited by the dynamical diffraction extinction depth of the polarization analyzer. The background intensity distribution in the XBI images is discussed elsewhere.^31^

## Conflicts of interest

There are no conflicts to declare.

## Supplementary Material

Supplementary information
