# Supplementary material for: Spatially resolved mapping of phase transitions in liquid-crystalline materials by X-ray birefringence imaging
Source: Chem Sci. 2019 Jan 2;10(10):3005–11. doi: 10.1039/c8sc05285a (PMC6430193; doi:10.1039/c8sc05285a)
Supplement: Supplementary file 1 [file SC-010-C8SC05285A-s001.pdf]

Electronic Supplementary Material (ESI) for Chemical Science.
This journal is © The Royal Society of Chemistry 2019

Electronic Supplementary Information

Spatially Resolved Mapping of Phase Transitions in Liquid-Crystalline Materials by X-ray Birefringence Imaging

Yating Zhou,[1] Rhian Patterson,[1,2] Benjamin A. Palmer,[1,3] Gregory R. Edwards-Gau,[1] Benson M. Kariuki,[1] N. S. Saleesh Kumar,[4] Duncan W. Bruce,[4] Igor P. Dolbnya,[2] Stephen P. Collins,[2] Andrew Malandain,[2] Kenneth D. M. Harris[1]*

1 School of Chemistry, Cardiff University, Park Place, Cardiff CF10 3AT, Wales

2 Diamond Light Source, Harwell Science and Innovation Campus, Didcot, Oxfordshire OX11 0DE, England

3 Department of Structural Biology, Weizmann Institute of Science, Rehovot, 760001, Israel

4 Department of Chemistry, University of York, Heslington, York YO10 5DD, England

Additional Figures

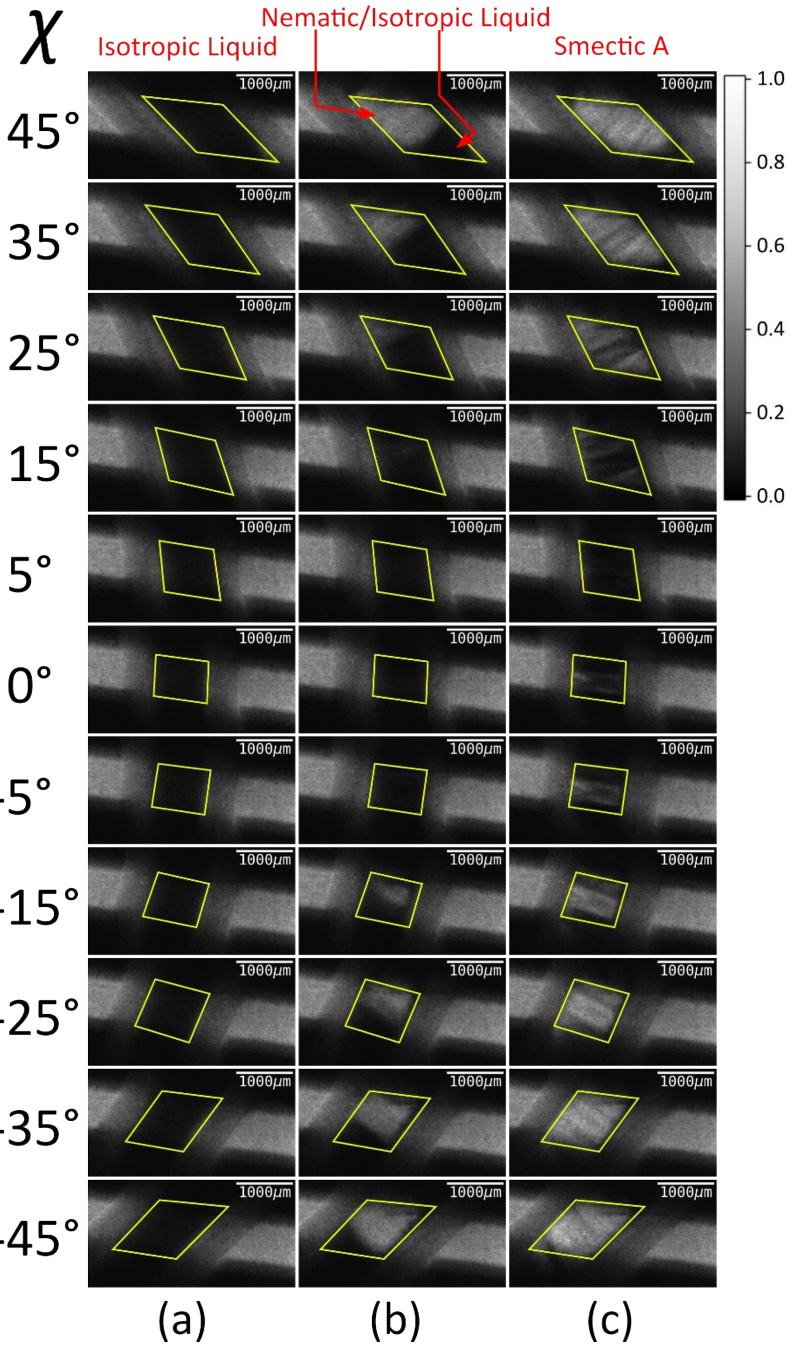

Figure S1: Selected XBI images recorded as a function of the orientation of the applied magnetic field (defined by angle χ) at the following temperatures: (a) 220 °C (isotropic liquid), (b) 214 °C (both nematic and isotropic liquid phases are present on account of a temperature gradient), and (c) 184 °C (smectic A phase). The scale of normalized X-ray intensity is shown on the right-hand side. The region of each XBI image representing the sample is defined by the yellow parallelogram. The data in this figure are from the same experiment as the data shown in Figure 4, but include a greater number of XBI images at different values of the angle χ compared to Figure 4.

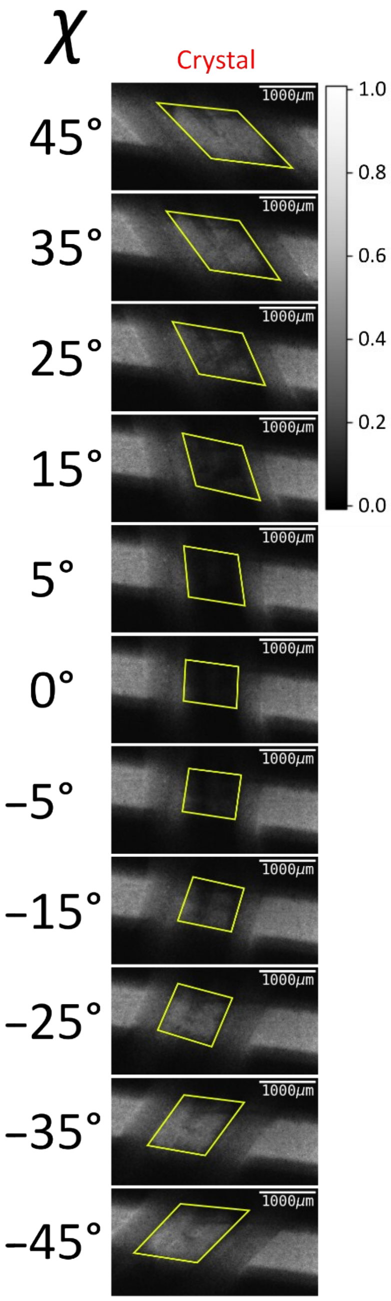

<u>Figure S2:</u> Selected XBI images recorded as a function of sample orientation angle χ for the crystalline phase at 100 °C. The scale of normalized X-ray intensity is shown on the right-hand side. The region of each XBI image representing the sample is defined by the yellow parallelogram. The data in this figure are from the same experiment as the data shown in Figure 4.

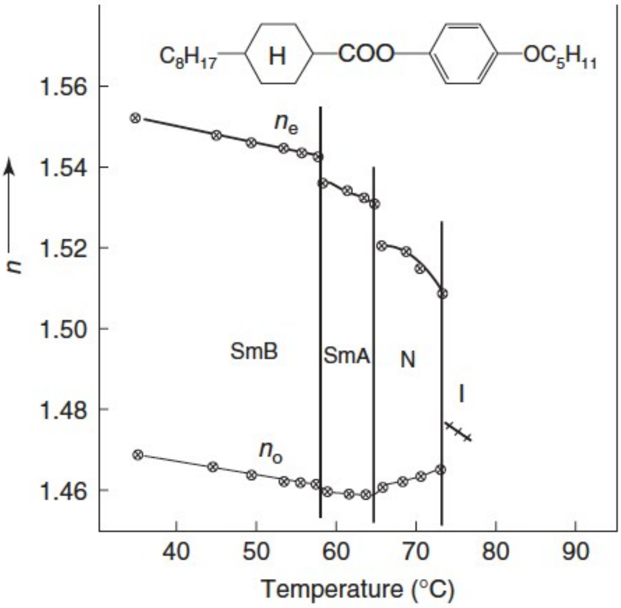

Figure S3: Values of refractive index (n_e and n_o) measured by optical microscopy as a function of temperature for a material (defined at the top of the figure) that exhibits the same sequence of phase transitions (Iso → N → SmA → SmB) as the material studied in the present work. [Data from: G. Pelzl in *Liquid Crystals*, Ed. H. Stegemeyer, Sternkopff, Darmstadt, 1994, p. 51. Copyright Wiley-VCH Verlag GmbH & Co. KGaA. Reproduced with permission.]
